# Polymorphisms in the Chicken Growth Differentiation Factor 9 Gene Associated with Reproductive Traits

**DOI:** 10.1155/2018/9345473

**Published:** 2018-09-19

**Authors:** Lingbin Liu, Zhifu Cui, Qihai Xiao, Haihan Zhang, Xiaoling Zhao, Yan Wang, Huadong Yin, Diyan Li, Qing Zhu

**Affiliations:** ^1^College of Animal Science and Technology, Southwest University, Chongqing, China; ^2^Farm Animal Genetic Resources Exploration and Innovation Key Laboratory of Sichuan Province, Sichuan Agricultural University, Chengdu Campus, Sichuan Province, China; ^3^Department of Animal and Poultry Sciences, Virginia Tech, Blacksburg, Virginia, USA

## Abstract

The aim of the study was to investigate* GDF9* gene polymorphisms and their association with reproductive traits in chicken using DNA sequencing. A total of 279 Dongxiang blue-shelled (DX) chickens and 232 Luhua (LH) chickens were used for validation. We detected 15 single nucleotide polymorphisms (SNPs): nine SNPs were previously unreported in chicken, two were missense mutations, and only three exhibited significant associations with reproductive traits. G.17156387C>T was significantly associated with age at first egg (AFE) and weight of first egg (WFE) in both breeds. Birds carrying the CC genotype exhibited higher AFE and WFE values than those with the TT genotype. The SNP g.17156427A>G exhibited an association with egg weight at 300 days of age (EWTA) in DX but not in LH chickens. The SNP g.17156703A>C affected the AFE and EN (total number of eggs at 300 days of age) in DX chickens. In addition, certain diplotypes significantly affected AFE, BWTA (body weight at 300 days of age), and EN in both breeds. RT-PCR results showed that the* GDF9* gene was highly expressed in stroma with cortical follicles (STR) and prehierarchal follicles. These results provided further evidence that the* GDF9* gene is involved in determining reproductive traits in chicken.

## 1. Introduction

In the modern poultry industry, reproductive traits of laying hens, such as age at first egg, egg number, and egg weight, are major factors of economic concern [1–4]. Studies on genes related to chicken reproductive traits are of great importance in terms of revealing genetic mechanisms affecting egg-laying performance and for breeding the laying hens with high productivity and quality. The candidate gene approach is a method used frequently for genetic dissection of complex and quantitative traits [5–9]. This method is based on using information regarding physiological and biochemical processes in the organism to select related genes and explore their relationship with phenotypes [3, 6]. Several crucial genes in livestock and poultry have been identified using this approach, such as the sex-linked dwarf gene in chicken [10], insulin-like growth factor 2 (*IGF2*) in swine [11], growth differentiation factor 8 (*GDF8*) in sheep [12], and diacylglycerol O-acyltransferase (*DGAT1*) in cattle [13, 14].

Growth differentiation factor 9 (*GDF9*), involved in growth and differentiation and secreted by oocytes, belongs to the transforming growth factor *β* superfamily and plays a critical role in ovarian follicular development and ovulation rate in mammals [15]. Further studies have shown that* GDF9* regulated hyaluronic acid synthesis, gonadotrophin-stimulated progesterone production, cumulus expansion, and the maintenance of an optimal oocyte microenvironment through synergistic action along with* BMP15* (bone morphogenetic protein 15) [16–18]. Besides granulosa cells closely adjacent to oocytes in some primates, the* GDF9* gene is exclusively expressed in oocytes within the ovary of most animals [19–23]. Recently, several studies have suggested that* GDF9* polymorphisms are associated with animal reproductive performance. Dag et al. [9] detected that a missense mutation (c.1111G>A) in* GDF9* causing a Val→Met substitution was significantly associated with litter size in sheep. The* FecG*^E^ allele of a novel* GDF9* polymorphism significantly increased ovulation rate and prolificacy in sheep [24]. Additionally,* GDF9* polymorphisms have also shown significant correlations with high prolificacy in goats [8, 25], the number of transferable embryos and ova in cows [26], and sperm quality traits in bulls [27]. However, most studies implicating effects of* GDF9* on reproductive performance have been conducted in mammals, and there are limited data regarding its role in the reproductive system of poultry.

Thus, we hypothesized that the* GDF9* gene was involved in the reproductive traits of laying hens. Single nucleotide polymorphisms (SNPs) are DNA sequence polymorphisms resulting from variation of a single nucleotide at the genomic level, including insertions, deletions, transversions, and transitions. SNPs comprise the latest generation of genetic markers and are often applied in animal breeding programs owing to their advantages such as high frequency, stability, and ease of genotyping [6, 28, 29]. To validate our hypothesis, in this study, we identified chicken* GDF9* SNPs via pooled DNA sequencing and investigated these SNPs association with reproductive traits in Luhua chicken and Dongxiang blue-shelled chicken. Furthermore, we analyzed expression levels of* GDF9 *mRNA in different chicken tissues and different breeds using quantitative real-time PCR.

## 2. Materials and Methods

### 2.1. Chicken Populations and Phenotypic Traits

Two chicken populations, Luhua chickens (LH, N = 232) and Dongxiang blue-shelled chickens (DX, N = 279), were used to assess the relationships between screened SNPs and reproductive traits. LH chickens are commercial egg-layers originating from the American Plymouth Rock and exhibit high egg productivity [4, 30]. DX chicken is an indigenous chicken breed from China that lays blue-shelled eggs with low egg productivity [31, 32]. Blue-shelled eggs are gaining popularity among consumers in some countries due to their high protein and low cholesterol contents [32]. Birds were reared in the poultry farm of Sichuan Agricultural University (Sichuan, China), and feed protocols and conditions followed those in previous research [33]. Six reproductive traits that are conventional selection indices in breeding programs for laying hens [3, 16, 34] were measured: age at first egg (AFE), body weight at first egg (BWFE), weight of first egg (WFE), body weight at 300 days of age (BWTA), egg weight at 300 days of age (EWTA), and total number of eggs at 300 days of age (EN). Blood samples were collected from the wing veins of all chickens. Genomic DNA was isolated using the phenol-chloroform extraction method following standard procedures [35], dissolved in TE buffer, and stored at –20°C. All animal care and experimental procedures were approved by the Institutional Animal Care and Use Committee of Sichuan Agricultural University (No. YYS130125). All research work was conducted in strict accordance with the Sichuan Agricultural University (SAU) Laboratory Animal Welfare and Ethics guidelines [36].

### 2.2. SNP Discovery and Genotyping

Discovery of* GDF9* gene polymorphisms and their association with reproductive traits in chickens was based on pooled DNA sequencing. Five primer pairs (P1–P5, Table 1) for amplifying and sequencing were designed with Primer Premier 5.0 (PREMIER Biosoft International, CA, USA) using the complete DNA sequence of the* GDF9* gene (Genbank accession number: NC_006100.4). Firstly, a pooled DNA sample containing 80 ng DNA from 60 chickens (30 birds from each breed) was constructed to identify SNPs. SNPs in PCR products were identified on the basis of multiple peaks at the same base position in sequencing results. Next, primer pairs producing SNP-containing PCR products were amplified using DNA from all birds (232 LH chickens and 279 DX chickens). Finally, eliminating low frequency SNPs (< 1%), we further investigated the relationship between these SNPs and reproductive traits in LH chickens and DX chickens.

All PCR amplifications were carried out using a PT-100 thermal cycler (MJ Research, USA) in a volume of 50 *μ*L, including 12 *μ*L of distilled H_2_O, 25 *μ*L of 2× Taq PCR Master Mix (Tiangen, China), 4 *μ*L of each primer (10 nmol/L), and 80 ng DNA template. PCR cycling conditions were as follows: 95°C for 5 min followed by 35 cycles of 95°C for 40 s, 51–59°C (depending on the primer pair, Table 1) for 40 s, and 72°C for 40 s and a full extension cycle at 72°C for 8 min. All PCR products were purified and sequenced using an ABI 3730 automated sequencer by a commercial sequencing facility (Sangon, Shanghai, China). All sequences were visually inspected, edited, assembled, and aligned using the software DNASTAR (DNASTAR Inc., USA). Haploview [37] was used to estimate linkage disequilibrium among SNP pairs. Pearson's chi-square test was used to determine whether polymorphisms in the sample population were under Hardy-Weinberg equilibrium. Haplotypes were constructed using the software PHASE [38].

### 2.3. Total RNA Isolation and Quantitative Real-Time PCR (qPCR)

Six 30-week-old birds were randomly selected from among the DX and LH egg-laying hens and then euthanized by cervical dislocation to collect the following ovarian tissues: stroma with cortical follicles < 1 mm in diameter (STR), prehierarchical follicles including white follicles (WF, 1–4 mm in diameter), yellowish follicles (YF, 4–8 mm in diameter) and small yellow follicles (SYF, 8–12 mm in diameter), and hierarchical follicles F1–F6 (with the largest denoted as F1, the second largest as F2, etc.). All samples were submerged in RNAlater (Invitrogen, Carlsbad, CA) overnight at 4°C and then stored at −80°C [39].

Total RNA was extracted from tissue samples with TRIzol RNA extraction reagent (TaKaRa Biotechnology Co. Ltd., Dalian, China). The concentration of total RNA was measured, its purity was validated with spectrophotometry at 260/280 nm, and its integrity was evaluated by 2% agarose gel electrophoresis. The first strand of cDNA was synthesized from 1 *µ*g total RNA using the PrimeScript RT Reagent Kit Perfect Real-Time (TaKaRa), according to the manufacturer's protocol. Real-time PCR primers were designed using the partial mRNA sequence of the* GDF9 *gene (Genbank accession number: NM_206988.2) without SNP sites using Primer Premier 5.0 (F: 5′–TACGCCACCAAGGAGGGAA–3′, R: 5′–AGCAAATCCACCGAGTGAAAGT–3′). The *β*-actin gene (Genbank accession number: NM_205518.1) was used as the endogenous control for normalization, and the primer sequences were as follows: forward primer (5′–GAGAAATTGTGCGTGACATCA–3′) and reverse primer (5′–CCTGAACCTCTCATTGCCA–3′). A total volume of 15 *μ*L in each reaction included 7.5 *μ*L of 2× SYBR Green SuperMix (Bio-Rad, Inc., Hercules, CA, USA), 0.6 *μ*L each of 10 *μ*M forward and reverse primers, 1 *μ*L of cDNA, and 5.3 *μ*L of RNase-free water. Real-time PCR reactions were performed in triplicate on a CFX96 qPCR system (Bio-Rad). The cycling conditions were as follows: 95°C for 30s, followed by 40 cycles of 95°C for 5s, a variable annealing temperature (*β*-actin: 61°C, and* GDF9*: 64.5°C) for 30s, and 72°C for 10s. The melting curve was determined and analyzed at 65-95°C. The amplification efficiencies of target genes ranged from 95% to 105%.

### 2.4. Statistical Analysis

Associations between* GDF9* polymorphisms and reproductive traits and expression levels were evaluated using SAS 9.0 (SAS Institute Inc., Cary, NC, USA). The relationship between polymorphisms and reproductive traits in each population was modeled as follows: Y_ij_ = *µ* + G_i_ + B_j_ + (GB)_ij_ + e_ij_, where Y_ij_ is the observation value for the trait; *µ* is the population mean of the trait; G_i_ is the fixed effect of the genotype or diplotype; B_j_ is the fixed effect of the breed; (GB)_ij_ is the interaction effect of the genotype or diplotype based on breed; and e_ij_ is the residual error. The quantification of relative mRNA expression was performed using the 2^-ΔΔCt^ method. Statistical significance was determined by the general linear model and further analyzed using Tukey's multiple comparison test. Results are presented as least square means ± SEM, and differences were considered significant at* P* < 0.05.

## 3. Results

### 3.1. *GDF9* SNP Identification

**Figure 1:**
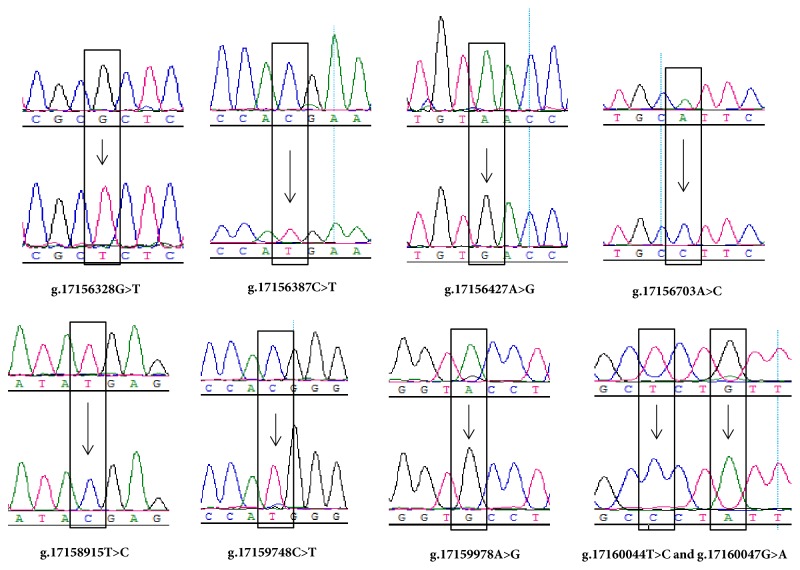
Nine previously unreported SNPs in chicken* GDF9* gene. Arrows indicate mutation sites.

The chicken* GDF9* gene is located on chromosome 13 and comprises two exons and one intron. We amplified and sequenced all exons, and parts of the intron and untranslated regions (UTR) of chicken* GDF9 *gene (Table 1). A total of 15 SNPs were detected by performing DNA sequencing of all chickens (Table 2) and included nine SNPs previously unreported in chicken, as shown in Figure 1. Among these polymorphic sites, three SNPs were located in the promoter region, one in exon 1, two in exon 2, and nine in the 3′ UTR. Among the exonic SNPs, two (g.17156703A>C and g.17158286A>G) were missense mutations, resulting in the amino acids substitutions of Ile→Leu and Ile→Val, respectively. Two SNPs (g.17159637T>G and g.17159638T>C) were found to be completely linked (*D*′ = 1.0, and* r*^2^ = 1.0); all other pairs of SNPs showed low LD values (*D*′ = 0.081~0.316, and* r*^2^ = 0.005~0.278).

The results of association analysis in all chickens showed that g.17156387C>T, g.17156427A>G, and g.17156703A>C were significantly associated with some reproductive traits (*P < 0.05*) (Table 3). Meanwhile, no significant associations were detected between other SNPs and any of the chicken reproductive traits (Table 3). Therefore, the following analysis including determining genotype frequency and performing detailed association analysis were only performed for the three SNPs.

### 3.2. Relationships between Genotypes and Reproductive Traits

**Figure 2:**
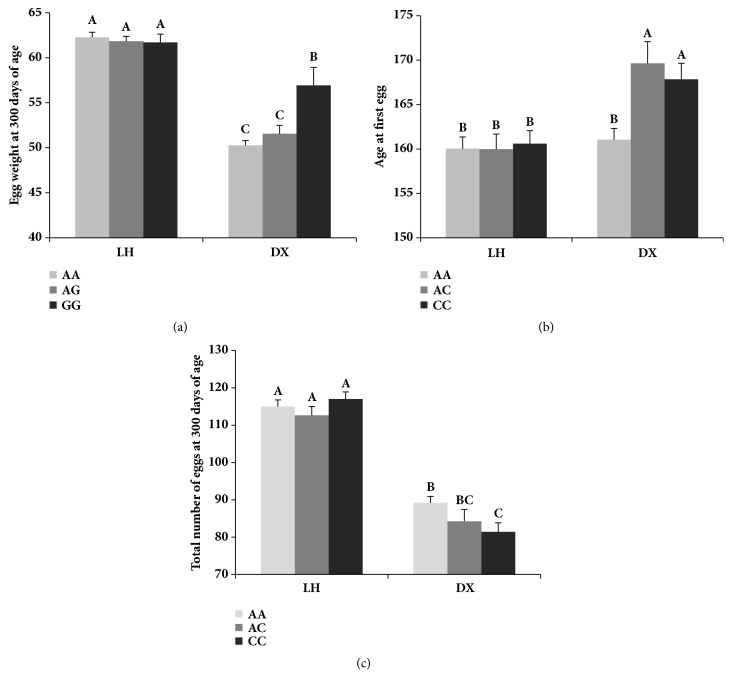
Effect of interactions between genotype and breed on (a) egg weight at 300 days of age (EWTA) in the case of the SNP g.17156427A>G and (b) age at first egg (AFE) and (c) total number of eggs at 300 days of age (EN) in the case of the SNP g.17156703A>C.

Results of genotype frequency analysis of the three SNPs were summarized in Table 4. Genotype frequency of these SNPs showed similar patterns, with the frequency of homozygous mutations being the lowest. Next, we evaluated the effect of the SNP g.17156387C>T (Table 5). The polymorphism of g.17156387C>T was significantly associated with AFE (0.04) and WFE (0.03). Birds carrying the CC genotype exhibited higher AFE and WFE values than those with the TT genotype. As shown in Table 6, the SNP g.17156427A>G only exhibited an association with EWTA (*P* = 0.03), and there was an interaction (*P* = 0.008) of genotype and breed where the genetic effect was associated with EWTA in DX but not LH chickens. Homozygous laying hens presenting the GG genotype have shown a significant increase in EWTA (56.92 g) compared to birds with the AA (50.27 g) and AG (51.56 g) genotypes in DX chickens (*P *< 0.05) (Figure 2(a)). The final* GDF9* SNP to be assessed was g.17156703A>C in exon 1 which showed significant effects on AFE (0.01) (Table 7). Some interactions of genotype and breed were also found in AFE (*P* = 0.02) and EN (*P* = 0.04). The polymorphism had a significant genetic effect on AFE and EN in DX hens (*P* < 0.05); birds with the AA genotype had lower AFE and higher EN values compared to those with other genotypes (Figures 2(b) and 2(c)). Meanwhile, this SNP had no effect on any of reproductive traits in LH chickens (Figure 2).

### 3.3. Relationships between Diplotypes and Reproductive Traits

A total of 19 diplotypes were identified in the present study, and eight diplotypes with frequencies more than 0.02 were selected from each breed to further evaluate associations with chicken reproductive traits (Table 8). The results showed that certain diplotypes significantly affected AFE (0.01), BWTA (0.04), and EN (0.04). Birds with the diplotype D2 (CCAAAC) exhibited the highest EN value, and those with D7 (CTAAAA) had the lowest AFE and BWTA values (Table 9). In addition, there were several differences between the two breeds with respect to all reproductive traits (Tables 5–7 and 9). BWFE, WFE, BWTA, EWTA, and EN were greater in LH than in DX chickens (*P* < 0.05), whereas AFE was greater in DX than in LH chickens (*P* < 0.05).

### 3.4. Expression of* GDF9* in Reproductive Tissues

**Figure 3:**
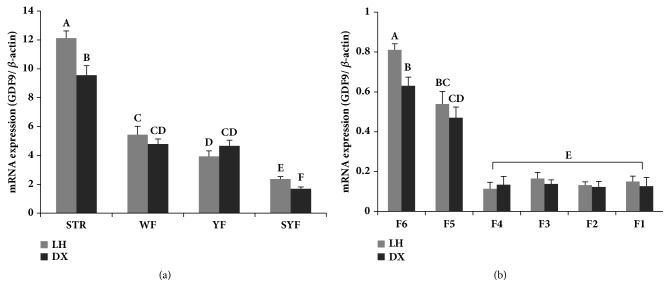
Relative expression of the* GDF9* gene in the ovaries of two chicken breeds. LH = Luhua chickens, DX = Dongxiang blue-shelled chickens, STR = stroma with cortical follicles, WF = white follicles, YF = yellowish follicles, SYF = small yellow follicles, and F1–F6 = hierarchical follicles F1–F6. Results are expressed as least square means ± standard errors. Different letters indicate significantly differences (*P *< 0.05).

In both breeds, the* GDF9* mRNA was most abundant in the STR, and all prehierarchical follicles (WF, YF, and SYF) exhibited higher expression of the* GDF9* gene than the hierarchical follicles (F1–F6). Moreover,* GDF9* mRNA expression was significantly higher in the STR, SYF, and F6 of LH chickens than in the corresponding tissues of DX chickens (*P* < 0.05) (Figure 3).

## 4. Discussion

### 4.1. Polymorphisms in the GDF*9* Gene

Numerous studies have shown that* GDF9* is a candidate gene involving reproductive performance and plays a critical role in animal reproductive biology [40–43]. However, the majority of these reports on the relationship between* GDF9* polymorphisms and reproductive traits have focused on swine, sheep, and goats. Zhang et al. [7] found 12 polymorphisms including three SNPs in coding regions and one 314-bp indel in the noncoding region of the swine* GDF9* gene. Hanrahan et al. [40] reported eight SNPs in the* GDF9* coding region through direct sequencing of Cambridge and Belclare sheep DNA, three of which were nonsense mutations and four of which were G>A mutations. Dong et al. [44] detected four SNPs in the coding sequence of goat* GDF9*, comprising C183A, C719T, A959C, and G1189A. Aside from C183A, these SNPs all resulted in a change in the encoded amino acid. In the present study, we sequenced the complete coding regions of the chicken* GDF9* gene and detected 15 SNPs including three located in the promoter region, one in exon 1, two in exon 2, and nine in the 3′ UTR. Among these, nine SNPs were previously unreported in chicken; two coding sequence SNPs were missense mutations, resulting in the amino acids substitutions of Ile→Leu and Ile→Val, respectively. These results significantly broaden our current understanding of* GDF9* polymorphisms underlying the reproductive systems of laying hens.

### 4.2. Association between GDF*9* and Chicken Reproduction Traits

Several different types of mutations and polymorphisms in* GDF9* have been reported to cause significant negative effects inducing infertility, ovulation impairment, and susceptibility to premature ovarian failure in some mammalian species [40, 42, 45]. In addition, positive effects on the ovulation rate in sheep and cows have been reported [24, 26, 46], and an increase in occurrence of dizygotic twins was reported in humans [47]. In this study, three mutations were found to have effects on reproductive traits. There are several possible mechanisms underlying this relationship. Promoter regions specifically identify and bind to transcription factors that recruit RNA polymerase, controlling the initiation of transcription and gene expression [48]. The two mutations located in the* GDF9* promoter region could influence transcriptional efficiency and subsequently alter the gene expression level. In the case of the SNP g.17156703A>C, it caused the amino acid substitution of Ile→Leu, which may subsequently result in a change of function in the* GDF9* protein.

In our study, BWFE, WFE, BWTA, EWTA, and EN were greater in LH than in DX chickens, whereas AFE was greater in DX than in LH chickens. This provided valuable evidence for differences in reproductive traits between the two breeds. We performed further association analyses and found that three SNPs affected several reproductive traits in DX hens, whereas only g.17156387C>T was significantly associated with two reproductive traits (AFE and WFE) in LH chickens. These results indicate that* GDF9* polymorphisms exert relatively stronger effects in DX than in LH chickens. Compared with LH chickens, DX chickens possess enormous potential for selective breeding because of their low productivity [30–32]. Thus, when major mutations related to reproductive traits are positively selected, DX chickens may experience greater relative genetic improvement.

Interestingly, no SNPs were associated with BWTA in either breeds or with EN in LH chickens, which are major traits of laying hens [3], but the integrated diplotype derived from the three SNPs significantly affected the two traits. A possible reason for this is positive synergy among these mutations, which may have small effects individually. This finding is consistent with previous observations of Cao et al. [49], who found that combined genotypes had more significant effects on body weight in chicken than individual genotypes did. In DX chickens, we found that the mutations g.17156387C>T, g.17156427A>G, and g.17156703A>C had significant effects on partial reproductive traits, including AFE, WFE, BWTA, EWTA, and EN. These results are similar to those of some previous reports showing that* GDF9* polymorphisms were associated litter size in goat [50], ovulation rate and prolificacy in sheep [24], and superovulation performance in cows [26]. Several studies are in agreement that diplotype analysis provides more information into the complex relationships between phenotypes and DNA variation [6, 51]. In the present study, we found that certain diplotypes significantly affected AFE, BWFE and EN in laying hens. Birds with the diplotype D2 exhibited the highest EN value, and those with D7 had the lowest AFE and BWTA values. The diplotype analysis found different effect sizes of the three mutations in the chicken* GDF9* gene with respect to reproductive traits. In addition, we found that laying hens with an earlier AFE had a higher EN. These results are consistent with a negative correlation between egg number and age at first egg often observed in quantitative genetics studies [1].

### 4.3. Expression of the* GDF9* Gene in Chicken Reproductive Tissues

The* GDF9* gene is expressed in human, rodent, and ruminant oocytes and is an essential factor that regulates development of ovarian follicles [17, 52, 53]. However, the expression pattern of* GDF9* differs across species. For example,* GDF9* expression was first reported in the primordial follicles of possum, hamsters, sheep, and cattle [19–21] and in the primary follicles of humans, mice, and rats [22, 23]. In this study,* GDF9* mRNA was found to be expressed in the STR, WF, YF, SYF, and all hierarchical follicles in chickens. These data indicate that* GDF9* also plays an important role in ovary function and follicle development in chicken. Chicken* GDF9* mRNA was most abundant in the STR and was relatively highly expressed in the WF and YF, consistent with previous studies in mammals, implying that* GDF9* is responsible for early development and differentiation of the ovarian follicles [41, 43, 54]. These results indicated that* GDF9* mainly affected the prehierarchal follicles in chicken. Yan et al. [17] showed that high expression level of* GDF9* is one of the important conditions for maintaining the growth of a large number of ovarian follicles. Intriguingly,* GDF9* expression was higher in the STR, SYF, and F6 of LH chickens than in DX counterparts. These results imply that the elevated expression of* GDF9* may be related to high reproductive performance in LH chickens.

## 5. Conclusion

In this study, we detected 15 SNPs in the chicken* GDG9* gene. Among these, nine SNPs were previously unreported in chicken; two (g.17156703A>C and g.17158286A>G) were missense mutations, resulting in the amino acids substitutions of Ile→Leu and Ile→Val, respectively; and three showed significant associations with partial reproductive traits. G.17156387C>T was significantly associated with AFE and WFE in both breeds. Birds carrying the CC genotype exhibited higher AFE and WFE values than those with the TT genotype. The SNP g.17156427A>G exhibited an association with EWTA in DX chickens but not LH chickens. This SNP g.17156703A>C had significant genetic effects on AFE and EN in DX hens; birds with the AA genotype had lower AFE and higher EN values compared to those with other genotypes. In addition, certain diplotypes significantly affected AFE, BWTA, and EN in both breeds.* GDF9* was found to be highly expressed in the STR and all prehierarchal follicles, and its expression was increased in the STR, SYF, and F6 of LH chickens compared to the corresponding tissues of DX chickens. These results broadened our current understanding of the chicken* GDF9* gene underlying reproductive systems and indicated that the GDF9 gene was involved in determining reproductive traits in chicken, but further studies are necessary for functional validation.

## Figures and Tables

**Table 1:** Primer pairs used to screen the *GDF9* gene for polymorphisms.

Primer pairs name	Primer sequences (5′–3′)	Binding regions	Product size (bp)	Annealing Tm (°C)	Chr. position
P1	F: GAAGCCGTAAGATGTGAA	Partial promoter region and exon 1, 5′UTR	701	51.5	17156010–17156720
	R: GGAAGAAAGCCAGTGAAT				
P2	F: CCTGAGAAGCAGCGTTTG	Exon 1, partial intron 1	796	58.7	17156430–17157225
	R: CAGCAGCCTCCACATTTT				
P3	F: ATTGGTTTCTTCTGCTGCTT	Exon 2, partial intron 1	882	58	17157996–17158877
	R: CATAACGGTGCCCGACTA				
P4	F: AACACGCAGGGCAAAAGG	Exon 2	971	54.2	17158631–17159601
	R: AAGTCCCGACCAAAGCAG				
P5	F: TCTGGGAAAAGAGGAAAG	Partial 3′UTR	711	46	17159411–17160111
	R: GTCTGAAATGGGTTGGTG				

**Table 2:** Summary of variations in the chicken *GDF9 *gene.

Primer pairs no.	Variations	Chr. position	Gene region	Amino acid position	Function
P1	g.17156328G>T	17156328	Promoter region		
P1	g.17156387C>T	17156387	Promoter region		
P1	g.17156427A>G	17156427	Promoter region		
P2	g.17156703A>C	17156703	Exon 1	15	Missense
P3	g.17158286A>G	17158286	Exon 2	198	Missense
P4	g.17158915T>C	17158915	Exon 2	407	Synonymous
P4	g.17159060T>C	17159060	3′ UTR		
P5	g.17159524A>T	17159524	3′ UTR		
P5	g.17159575C>T	17159575	3′ UTR		
P5	g.17159624T>G	17159624	3′ UTR		
P5	g.17159625T>C	17159625	3′ UTR		
P5	g.17159748C>T	17159748	3′ UTR		
P5	g.17159978A>G	17159978	3′ UTR		
P5	g.17160044T>C	17160044	3′ UTR		
P5	g.17160047G>A	17160047	3′ UTR		

UTR means untranslated region.

**Table 3:** Association of *GDF9* polymorphisms with reproductive traits in all chickens.

Polymorphism	Traits (*P*-value of significance test)					
	AFE	BWFE	WFE	BWTA	EWTA	EN
g.17156328G>T	0.29	0.08	0.15	0.05	0.09	0.32
g.17156387C>T	0.04∗	0.16	0.03∗	0.78	0.49	0.42
g.17156427A>G	0.6	0.58	0.54	0.98	0.03∗	0.69
g.17156703A>C	0.01∗	0.41	0.82	0.14	0.78	0.16
g.17158286A>G	0.60	0.44	0.15	0.13	0.21	0.13
g.17158915T>C	0.36	0.47	0.36	0.08	0.21	0.15
g.17159060T>C	0.52	0.60	0.47	0.47	0.29	0.47
g.17159524A>T	0.07	0.55	0.12	0.12	0.10	0.30
g.17159575C>T	0.20	0.73	0.11	0.13	0.19	0.06
g.17159624T>G	0.09	0.20	0.43	0.61	0.11	0.20
g.17159625T>C	0.10	0.20	0.08	0.41	0.24	0.52
g.17159748C>T	0.46	0.08	0.09	0.14	0.23	0.08
g.17159978A>G	0.20	0.13	0.05	0.30	0.08	0.30
g.17160044T>C	0.67	0.08	0.28	0.41	0.14	0.15
g.17160047G>A	0.31	0.18	0.51	0.22	0.26	0.13

AFE = age at first egg, BWFE = body weight at first egg, WFE = weight of first egg, BWTA = body weight at 300 days of age, EWTA = egg weight at 300 days of age, and EN = total number of eggs at 300 days of age.

∗ Significant association (*P* < 0.05).

**Table 4:** Genotype frequencies in three SNPs of *GDF9*.

SNP	Genotype	ALL	LH	DX
g.17156387C>T	CC	0.62(317)	0.81(188)	0.46(129)
	CT	0.33(166)	0.17(38)	0.46(128)
	TT	0.05(28)	0.02(6)	0.08(22)

g.17156427A>G	AA	0.60(304)	0.41(94)	0.75(210)
	AG	0.32(165)	0.44 (102)	0.23 (63)
	GG	0.08(42)	0.15(36)	0.02(6)

g.17156703A>C	AA	0.37(188)	0.32(73)	0.41(115)
	AC	0.45(231)	0.45(105)	0.45(126)
	CC	0.18(92)	0.23(54)	0.14(38)

LH means Luhua chickens (N = 232), DX means Dongxiang blue-shelled chickens (N = 279), and ALL means the sum of the number of LH and DX chickens (N = 511).

**Table 5:** Association analysis between *GDF9* g.17156387C>T genotypes and reproductive traits.

	AFE (days)	BWFE (g)	WFE (g)	BWTA (g)	EWTA (g)	EN (count)
Genotype						
CC	165.85±0.96^a^	1799.65±27.46	44.13±0.67^a^	1722.41±19.19	58.33±0.61	98.53±1.48
CT	161.97±1.51^b^	1482.07±42.82	35.72±1.17^b^	1594.05±35.9	53.11±0.8	103.81±2.56
TT	162.13±2.04^ab^	1566.25±189.39	38.33±3.52^ab^	1650.63±127.14	55.61±2.18	102.63±5.28
P Value	0.04	0.16	0.03	0.78	0.49	0.42

Breed						
LH	161.31±0.84^b^	2038.52±34.38^a^	46.77±1.45^a^	1869.7±44.75^a^	61.93±0.94^a^	112.65±2.84^a^
DX	165.61±1.21^a^	1305.22±26.25^b^	34.08±1.11^b^	1503.48±34.21^b^	50.65±0.72^b^	84.91±2.17^b^
P Value	0.008	<0.0001	<0.0001	<0.0001	<0.0001	<0.0001

G x B						
P Value	0.06	0.1	0.47	0.39	0.54	0.64

AFE = age at first egg, BWFE = body weight at first egg, WFE = weight of first egg, BWTA = body weight at 300 days of age, EWTA = egg weight at 300 days of age, and EN = total number of eggs at 300 days of age.

LH means Luhua chickens (N = 232), and DX means Dongxiang blue-shelled chickens (N = 279).

Results are expressed as least square means ± standard errors.

Different letters indicate significant differences (*P* < 0.05).

**Table 6:** Association analysis between *GDF9* g.17156427A>G genotypes and reproductive traits.

	AFE (days)	BWFE (g)	WFE (g)	BWTA (g)	EWTA (g)	EN (count)
Genotype						
AA	163.38±1.19	1616.7±34.69	43.75±0.84	1641.09±24.31	55.35±0.66^b^	98.45±1.89
AG	161.5±1.09	1793.75±40.97	42.97±1.03	1737.36±28.84	59.04±0.73^ab^	105.09±2.08
GG	162.36±2.11	1853.18±55	41.54±1.79	1734.09±43.04	60.83±1.16^a^	103.44±3.56
*P*-value	0.6	0.58	0.54	0.98	0.03	0.69

Breed						
LH	159.52±0.74^b^	1996.03±15.49^a^	46.96±0.67^a^	1815.86±18.21^a^	61.98±0.39^a^	114.59±1.18^a^
DX	166.51±1.41^a^	1336.56±15.48^b^	34.85±0.66^b^	1519.62±22.62^b^	50.86±0.41^b^	86.02±1.41^b^
*P*-value	0.0009	<0.0001	<0.0001	<0.0001	<0.0001	<0.0001

G x B						
*P*-value	0.49	0.31	0.99	0.49	0.008	0.44

AFE = age at first egg, BWFE = body weight at first egg, WFE = weight of first egg, BWTA = body weight at 300 days of age, EWTA = egg weight at 300 days of age, and EN = total number of eggs at 300 days of age.

LH means Luhua chickens (N = 232), and DX means Dongxiang blue-shelled chickens (N = 279).

Results are expressed as least square means ± standard errors.

Different letters indicate significant differences (*P* < 0.05).

**Table 7:** Association analysis between *GDF9* g.17156703A>C genotypes and reproductive traits.

	AFE (days)	BWFE (g)	WFE (g)	BWTA (g)	EWTA (g)	EN (count)
Genotype						
AA	160.57±0.85^b^	1635.1±38.09	40.89±1.04	1634.25±27.78	55.73±0.69	103.35±1.82
AC	163.52±0.69^a^	1780.47±51.69	42.57±1.33	1753.84±32.22	57.88±1.19	102.95±2.89
CC	163.95±1.24^a^	1754.09±42.14	41.95±0.88	1714.02±27.72	57.64±0.9	101.31±2.51
*P*-value	0.01	0.41	0.82	0.14	0.78	0.16

Breed						
LH	160.21±0.69^b^	1996.11±15.11^a^	46.83±0.64^a^	1816.94±19.42^a^	62.01±0.41^a^	114.89±1.16^a^
DX	164.25±1.18^a^	1343.39±19.07^b^	35.01±0.82^b^	1547.98±24.52^b^	50.01±0.52^b^	84.94±1.45^b^
*P*-value	<0.0001	<0.0001	<0.0001	<0.0001	<0.0001	<0.0001

G x B						
*P*-value	0.02	0.37	0.19	0.21	0.35	0.04

AFE = age at first egg, BWFE = body weight at first egg, WFE = weight of first egg, BWTA = body weight at 300 days of age, EWTA = egg weight at 300 days of age, and EN = total number of eggs at 300 days of age. LH means Luhua chickens (N = 232), and DX means Dongxiang blue-shelled chickens (N = 279).

Results are expressed as least square means ± standard errors.

Different letters indicate significant differences (*P* < 0.05).

**Table 8:** Diplotypes and their frequencies.

Diplotype	ALL	LH	DX
CCAAAA	0.2(104)	0.19(44)	0.22(60)
CCAAAC	0.06(30)	0.08(18)	0.04(12)
CCAACC	0.06(33)	0.05(12)	0.08(21)
CCAGAA	0.07(36)	0.1(24)	0.04(12)
CCAGAC	0.07(38)	0.14(32)	0.02(6)
CCAGCC	0.1(49)	0.15(34)	0.05(15)
CCGGAA	0.02(8)	0.03(8)	
CCGGAC	0.01(5)	0.01(2)	0.01(3)
CCGGCC	0.05(26)	0.11(26)	
CTAAAA	0.16(81)	0.05(12)	0.25(69)
CTAAAC	0.04(20)	0.01(2)	0.06(18)
CTAACC	0.04(20)	0.01(2)	0.06(18)
CTAGAA	0.03(13)	0.02(4)	0.03(9)
CTAGAC	0.01(5)	0.01(2)	0.01(3)
CTAGCC	0.05(27)	0.03(6)	0.08(21)
TTAAAA	0.02(8)	0.01(2)	0.02(6)
TTAAAC	0(2)	0.01(2)	
TTAACC	0.01(3)		0.01(3)
TTGGAA	0.01(3)		0.01(3)

LH means Luhua chickens (N = 232), DX means Dongxiang blue-shelled chickens (N = 279), and ALL means the sum of the number of LH and DX (N = 511).

**Table 9:** Association analysis between *GDF9* diplotypes and reproductive traits.

	AFE (days)	BWFE (g)	WFE (g)	BWTA (g)	EWTA (g)	EN (count)
Diplotype						
D1 (CCAAAA)	166.46±1.62^a^	1673.07±31.26	43.26±1.01	1687.3±22.26^ab^	55.66±0.66	99.38±1.95^ab^
D2 (CCAAAC)	159.35±3.15^bc^	1601.39±60.79	39.89±1.96	1627.36±43.29^bc^	55.38±1.29	107.21±3.79^a^
D3 (CCAACC)	165.73±2.91^ab^	1753.1±56.28	40.43±1.81	1768.57±40.08^a^	56.37±1.19	102.38±3.5^a^
D4 (CCAGAA)	160.75±3.02^abc^	1583.13±58.41	42.11±1.88	1626.67±41.6^bc^	58.01±1.24	106.67±3.64^a^
D5 (CCAGAC)	167.16±3.93^ab^	1785±75.87	41.97±2.45	1666.25±54.03^abc^	54.59±1.61	89.84±4.72^b^
D6 (CCAGCC)	162.23±2.67^abc^	1655±51.47	40.86±1.66	1657.06±36.65^bc^	55.49±1.09	102.76±3.21^a^
D7 (CTAAAA)	155.54±2.4^c^	1597.46±46.38	37.28±1.49	1593.77±33.03^c^	55.05±0.98	103.91±2.89^a^
D8 (CTAGCC)	162.19±3.62^abc^	1652.74±69.81	39.17±2.25	1626.19±49.72^bc^	56.43±1.48	99.5±4.35^ab^
*P*-value	0.01	0.19	0.08	0.04	0.66	0.04

Breed						
LH	158.4±1.33^b^	1982.45±18.26^a^	45.36±0.83^a^	1800.59±25.64^a^	61.97±0.54^a^	115.66±1.6^a^
DX	166.45±1.65^a^	1330.84±22.63^b^	35.89±1.02^b^	1524.63±31.78^b^	49.78±0.67^b^	87.01±1.98^b^
*P*-value	0.0002	<0.0001	<0.0001	<0.0001	<0.0001	<0.0001

D x B						
*P*-value	0.23	0.94	0.97	0.94	0.06	0.76

N = number, F = frequency, AFE = age at first egg, BWFE = body weight at first egg, WFE = weight of first egg, BWTA = body weight at 300 days of age, EWTA = egg weight at 300 days of age, and EN = total number of eggs at 300 days of age.

LH = Luhua chickens, DX = Dongxiang blue-shelled chickens.

Results are expressed as least square means ± standard errors.

Different letters indicate significant differences (*P* < 0.05).

## Data Availability

The data used to support the findings of this study are available from the corresponding author upon request.
