# Corrigendum to “Polymorphisms in the Chicken Growth Differentiation Factor 9 Gene Associated with Reproductive Traits”

**DOI:** 10.1155/2018/4963834

**Published:** 2018-11-28

**Authors:** Lingbin Liu, Zhifu Cui, Qihai Xiao, Haihan Zhang, Xiaoling Zhao, Yan Wang, Huadong Yin, Diyan Li, Qing Zhu

**Affiliations:** ^1^Farm Animal Genetic Resources Exploration and Innovation Key Laboratory of Sichuan Province, Sichuan Agricultural University, Chengdu Campus, Sichuan Province, China; ^2^College of Animal Science and Technology, Southwest University, Chongqing, China; ^3^Department of Animal and Poultry Sciences, Virginia Tech, Blacksburg, Virginia, USA

In the article titled “Polymorphisms in the Chicken Growth Differentiation Factor 9 Gene Associated with Reproductive Traits” [1], there was an error in the order of the first and second affiliations. The corrected affiliation list is shown above.

## References

[1] Liu L., Cui Z., Xiao Q. (2018). Polymorphisms in the Chicken Growth Differentiation Factor 9 Gene Associated with Reproductive Traits. *BioMed Research International*.

